# Stability Indicating HPLC Determination of Risperidone in Bulk Drug and Pharmaceutical Formulations

**DOI:** 10.1155/2011/124917

**Published:** 2011-10-10

**Authors:** Zarna R. Dedania, Ronak R. Dedania, Navin R. Sheth, Jigar B. Patel, Bhavna Patel

**Affiliations:** ^1^Department of Pharmaceutical Analysis, Veerayatan Institute of Pharmacy, Gujarat, Kutch, India; ^2^Department of Pharmaceutical Sciences, Saurashtra University, Gujarat, Rajkot, India; ^3^K. B. Institute of pharmaceutical Education and Research, Gandhinagar, India; ^4^CVM Institute for Degree Course in Pharmacy, New Vallabh Vidhyanagar, Anand, India

## Abstract

The objective of the current study was to develop a validated stability-indicating assay method (SIAM) for risperidone after subjecting it to forced decomposition under hydrolysis, oxidation, photolysis, and thermal stress conditions. The liquid chromatographic separation was achieved isocratically on a symmetry C18 column (5 **μ**m size, 250 mm × 4.6 mm i.d.) using a mobile phase containing methanol: acetonitrile (80 : 20, v/v) at a flow rate of 1 mL/min and UV detection at 280 nm. Retention time of risperidone was found to be 3.35 ± 0.01. The method was linear over the concentration range of 10–60 **μ**g/mL(*r* ^2^ = 0.998) with a limit of detection and quantitation of 1.79 and 5.44 **μ**g/mL, respectively. The method has the requisite accuracy, specificity, sensitivity, and precision to assay risperidone in bulk form and pharmaceutical dosage forms. Degradation products resulting from the stress studies did not interfere with the detection of Risperidone, and the assay is thus stability indicating.

## 1. Introduction

Risperidone, 4-[2-[4-(6-fluorobenzo[d]isoxazol-3-yl)-1-piperidyl] ethyl]-3-methyl-2,6-diazabicyclo[4.4.0]deca-1,3-dien-5-one an atypical antipsychotic drug used for the treatment of schizophrenia, the mixed and manic states associated with bipolar disorder, and irritability in children with autism [1]. Blockade of dopaminergic D2 receptors in the limbic system alleviates positive symptoms of schizophrenia such as hallucinations, delusions, and erratic behavior and speech. Blockade of serotonergic 5-HT_2_ receptors in the mesocortical tract causes an excess of dopamine, resulting in an increase in dopamine transmission and an elimination of core negative symptoms. It has high affinity for D_2_ dopaminergic receptors. It has actions at several 5-HT (serotonin) receptor subtypes. These are 5-HT_2C_, linked to weight gain, 5-HT_2A_, linked to its antipsychotic action and relief of some of the *extrapyramidal* side effects experienced with the typical neuroleptics through action at 5-HT_1A_. Like other 5-HT_2_ antagonists, risperidone also binds at alpha (1)-adrenergic receptors and, to a lesser extent, at histamine H1 and alpha (2)-adrenergic receptors [2]. Several analytical methods have been reported in the literature for the analysis of Risperidone from pharmaceutical dosage form. The techniques include chemiluminescence [3], RP HPLC [4], TLC [5], and visible spectrophotometry [6] and so forth forms. There are numerous methods to quantify Risperidone in biological fluid and human plasma, including HPLC combined with capillary electrophoresis [7], HPLC-DAD [8], HPLC-MS/ MS [9], RP-HPLC [10], LC-MS/MS [11–13], affinity capillary electrophoresis, and H_1_ NMR spectroscopy [14]. These methods [11–14] are complicated, costly and time consuming in comparison to a simple HPLC-UV method. Currently, most of the separations are performed by HPLC for reasons of robustness and familiarity of analysts with this technique. About specificity for HPLC method, peak purity tests may be useful to show that the analyte chromatographic peak is not attributable to more than one component. The previous published methods are not directly applicable for this issue and need more investigation for method development and validation. So, an approach was made to develop a simple, precise, accurate, specific, and robust stability- indicating HPLC-UV method for the quantitative determination of Risperidone in pharmaceutical dosage forms and applied to the assay of Risperidone in tablets and bulk form.

## 2. Experimental

### 2.1. Chemicals and Reagents

Risperidone working standard powder with 99.98% purity was kindly gifted by Tripada Pharmaceuticals, Ahmedabad, India and was used without further purification. RISPERDAL tablets containing 2 mg Risperidone as per labelled claim were obtained from local market. methanol, acetonitrile, and water were of HPLC grade, and sodium hydroxide, and hydrochloric acid, hydrogen peroxide were of analytical grade obtained from E. Merck (India) Ltd., Mumbai. All chemicals were at least of analytical grade and used as received.

### 2.2. HPLC Instrumentation and Conditions

The present work was carried out on isocratic high pressure liquid chromatography and consisted of pump (Cyberlab TM, USA) with universal loop injector (Rheodyne) of injection capacity 20 *μ*L. Detector consists of photodiode array detector; the reversed phase column used was RP-C18 (5 *μ*m size, 250 mm  ×  4.6 mm i.d.) at ambient temperature. Separation was achieved using a mobile phase consisting of methanol : acetonitrile (80 : 20, v/v) at a flow rate of 1 mL/min. The eluted compounds were monitored at 280 nm. The column was maintained at ambient temperature, and an injection volume of 20 *μ*L was used. The mobile phase was filtered through 0.45 micron membrane filter and ultrasonicated for 10 minutes prior to use. For analysis of forced degradation samples, the photodiode array detector was used in scan mode with a scan range of 200–400 nm. Peak homogeneity was expressed in terms of peak purity values and was obtained directly from spectral analysis report obtained using the instrument software.

### 2.3. Preparation of Stock and Standard Solutions

A 1 mg/mL stock solution of Risperidone was prepared in HPLC grade acetonitrile. Substock solution was prepared from stock solution by diluting 10 mL standard stock solution up to 100 mL to get 100 *μ*g/mL. Aliquots of the standard Substock solution of Risperidone were transferred into 10 mL volumetric flasks and the solutions were made up to volume with mobile phase to give final concentrations of 10, 20, 30, 40, 50, and 60 *μ*g/mL. Each 20 *μ*L standard solution was injected into the column after filtration using 0.2 micron membrane filter.

### 2.4. Preparation of Tablets for Assay

Twenty tablets were weighed, crushed, and mixed in a mortar and pestle. A portion of powder equivalent to the weight of one tablet was accurately weighed and transferred into 10 mL volumetric flask and made up to the volume with mobile phase and mixed well. Six of such solutions were prepared. The volumetric flasks were sonicated for 20 minutes to affect complete dissolution of the Risperidone, and the solutions were then made up to the volume with mobile phase. Suitable aliquots of solution were filtered through a 0.45 micron nylon filter. 2 mL of the filtered solution was transferred to a 10 mL volumetric flask and made up to the volume with mobile phase to yield concentration of Risperidone 40 *μ*g/mL in the range of linearity previously described.

### 2.5. Forced Degradation Studies of API

In order to determine the stability indication of the analytical method and assay, Risperidone tablets and Risperidone API powder were stressed under various conditions to conduct forced degradation studies [15]. Regulatory guidance in ICH Q2A, Q2B, Q3B, and FDA 21 CFR section 211 requires the development and validation of stability-indicating potency assays. Unfortunately, the current guidance documents do not indicate detailed degradation conditions in stress testing. However, the forced degradation conditions, stress agent concentration, and time of stress were selected based on trial and error method, so that the degradation of drug preferably remains between 10% and complete degradation. As Risperidone is practically insoluble in water and is readily soluble in methanol, methanol was used as cosolvent in all studies. All solutions of Risperidone used in forced degradation studies were prepared with final concentrations of 20 *μ*g/mL.

#### 2.5.1. Acid Degradation Studies

100 mg of Risperidone sample was taken into a 100 mL round bottom flask, 10 mL of 0.1 M hydrochloric acid solution was added, and contents were mixed well and kept for constant stirring for 12 hr at room temperature. 2 mL of this solution was taken in 100 mL volumetric flask and neutralized with 0.2 mL of 0.1 M sodium hydroxide and then diluted to 100 mL with diluent.

#### 2.5.2. Alkali Degradation Studies

100 mg of Risperidone sample was taken into a 100 mL round bottom flask, 10 mL of 0.1 M sodium hydroxide solution was added, and contents were mixed well and kept for constant stirring for 36 hr at room temperature. 2 mL of this solution was taken in 100 mL volumetric flask and neutralized with 0.2 mL of 0.1 M hydrochloric acid and then diluted to 100 mL with diluent.

#### 2.5.3. Oxidation

100 mg of Risperidone sample was taken in 100 mL round bottom flask, 10 mL of 3% hydrogen peroxide solution was added, and contents were mixed well at room temperature. After 4 hr, 2 mL of this solution was diluted to 100 mL with diluent.

#### 2.5.4. Temperature Stress Studies

1 g of Risperidone sample was taken into a petridish and kept in oven at 80°C for 24 hr. 10 mg of this sample was taken into a 100 mL volumetric flask, dissolved in diluent and diluted to volume with diluent. 2 mL of this solution was taken in 10 mL volumetric flask and then diluted to 10 mL with diluent.

#### 2.5.5. Photostability

1 g of Risperidone sample was taken in to a petridish and kept in photostability chamber 200 Wh/m^2^ in UV light and 1.2 million *l* × *h* in visible light for 36 hr. 10 mg of this sample was taken in to a 100 mL volumetric flask, dissolved in diluents, and diluted to volume with diluent. 2 mL of this solution was taken in 10 mL volumetric flask and then diluted to 10 mL with diluent.

## 3. Results and Discussion

### 3.1. HPLC Method Development and Optimization

A RP-C18 column (250 mm × 4.6 mm i.d., 5 *μ*m particle size) maintained at ambient temperature (25°C) was used for the separation and the method validated for the determination of Risperidone in pharmaceutical dosage forms. Initially mobile phase composed of n-hexane : ethanol : methanol (50 : 35 : 15, v/v/v) was tried, but analyte could not be resolved in 20 min run time. Then different mobile phase composition like 0.1% formic acid : acetonitrile (40 : 60, v/v); sodium dihydrogen phosphate buffer-acetonitrile (55 : 45, v/v) adjusted to pH 6.0 at 1.5 mL/min flow rate were tried, but poor resolution was obtained. So the addition of acetonitrile in above mobile phase and mobile phase composed of methanol : acetonitrile : 0.05 M potassium dihydrogen phosphate pH 7 (65 : 25 : 10 v/v) at 1.5 mL/min flow rate were tried but satisfactory peak was not observed. Then buffer solution was replaced with organic solvent to simplify the tediousness of preparation of the mobile phase. The mobile phase methanol : acetonitrile (60 : 40, v/v) was tried and get resolved peak of analyte and degradants. Improvement of peak shape and symmetry was done by changing different ratio and flow rate of methanol : acetonitrile : water. A satisfactory separation and peak symmetry for the drug and its degradation products were obtained with mobile phase consisting of methanol : acetonitrile (80 : 20,v/v) at 1 mL/min flow rate and ambient temperature.

### 3.2. Validation

The method was validated with respect to parameters including linearity, limit of quantitation (LOQ), limit of detection (LOD), precision, accuracy, specificity, robustness, solution stability, and system suitability.

#### 3.2.1. Linearity

The calibration curves constructed for Risperidone were linear over the concentration range of 10–60 *μ*g/mL. Peak areas of Risperidone were plotted versus Risperidone concentration and linear regression analysis performed on the resultant curve. Typically, the regression equation for the calibration curve was found to be *y* = 5203*x* + 8933 with 0.999 correlation coefficient.

#### 3.2.2. LOQ and LOD

The LOQ and LOD were determined based on signal-to-noise ratios, using an analytical response of 10 and three times of the background noise, respectively [16]. The LOQ and LOD were found to be 5.44 *μ*g/mL and 1.79 *μ*g/mL, respectively.

#### 3.2.3. Precision

Table 1 provides data obtained from the precision experiments. The R.S.D. values for intraday and interday precision were <1.04% and <1.22%, respectively, thereby indicating that the method was sufficiently precise. A similar qualitative separation of the drug was obtained even on analysis on a different chromatographic system on a different day, indicating that the method has sufficient intermediate precision.

#### 3.2.4. Accuracy

Percentage recovery was calculated from differences between the peak areas obtained for fortified and unfortified solutions. As shown from the data in Table 2, excellent recoveries were made at each added concentration of drug.

#### 3.2.5. Specificity

Specificity is the ability of the method to measure the analyte response in the presence of its degradation products. The method was found to be specific to the drug. Specificity was established by determination of purity of the drug peak using a PDA detector. Also, the resolution factor of the drug peak was determined with respect to the nearest resolving peaks.

#### 3.2.6. Robustness

The robustness was illustrated by getting the retention time, when mobile phase flow rate (±0.2 mL/min), organic solvent ratio (±5%), and column temperature (±2°C) were deliberately varied.

#### 3.2.7. Solution Stability

The solution stability of Risperidone in diluents was determined by leaving 0.1 mg/mL sample solution in a tightly capped volumetric flask at room temperature for 48 hr and measuring the amount for every 6 hr. The solution stability of Risperidone assay (in diluent) was determined by leaving 0.1 mg/mL Risperidone solution in tightly capped volumetric flasks at room temperature for 48 hr during which they were assayed at 6 hr intervals and compared the results with those obtained from freshly prepared solution. The mobile phase was prepared at the beginning of the study period and not changed during the experiment. The %RSD values for solution stability experiments were calculated and found to be 1.36% and 1.45% for purity and assay methods, respectively. All the samples were found to be stable up to 48 hr.

#### 3.2.8. System Suitability

The system suitability test was applied to a representative chromatogram to check the various parameters such as column efficiency, resolution, precision, and peak tailing. The result obtained is shown in Table 3. All these parameters were evaluated with the background of regulatory requirements, which suggests good chromatographic condition.

### 3.3. Stability Studies

**Figure 1:**
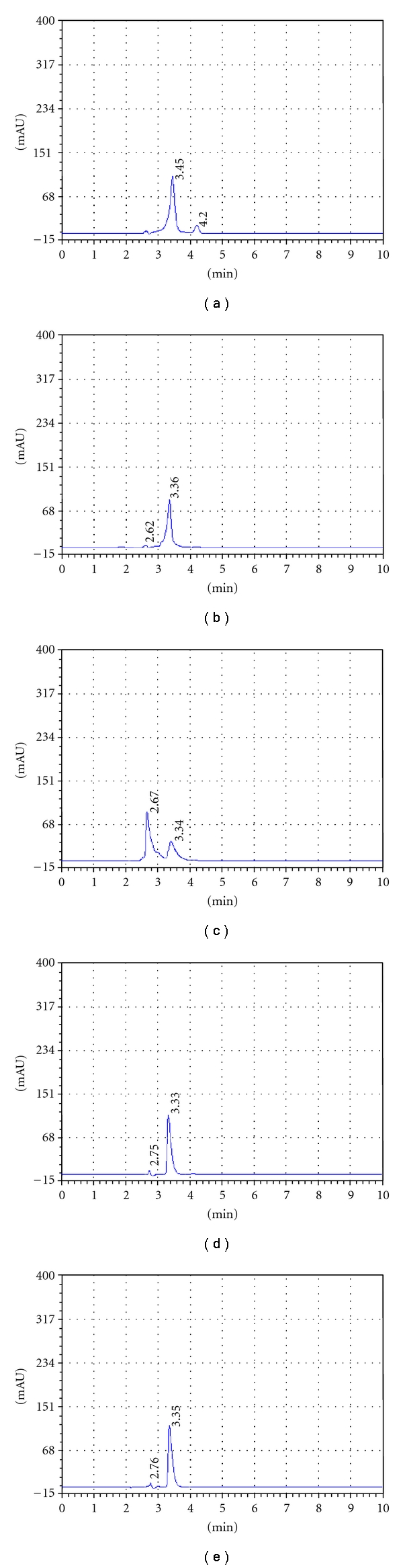
Typical HPLC chromatograms of: (a) acid hydrolysis-degraded active pharmaceutical ingredient (API), (b) base hydrolysis-degraded API, (c) oxidative degraded API, (d) thermal degrade API, and (e) photodegraded (API).

All stressed samples in both solid and solution state remained colorless. HPLC studies on Risperidone under different stress conditions using methanol : acetonitrile (80 : 20, v/v) as the solvent system suggested the following degradation behavior as shown in Figure 1. Summary of degradation was shown in Table 4.

#### 3.3.1. Acidic Condition

The drug gradually decreased with time on refluxing with 0.1 M HCl at 12 hr at room temperature, forming degradation products at RRT 4.20. At the end of 24 h, around 26.89% fall in drug peak area was observed, and fronting was observed in analyte peak.

#### 3.3.2. Degradation in Alkali

The drug was found to comparatively stable to alkaline hydrolysis. On refluxing the drug in 0.1 M NaOH for 36 hrs, around 17.53% of the drug was only degraded. Degradants product was appeared at RRT 2.62.

#### 3.3.3. Oxidative Conditions

The drug was highly labile to hydrogen peroxide (3%) at room temperature. After 4 hr, steep fall in the drug peak area was observed. Major degradants products were appeared at RRT 2.67, and risperidone was degraded up to 68.54%. At the end of 6 hr, almost complete degradation of the drug was observed with the corresponding rise in the major degradation peak.

#### 3.3.4. Thermal Degradation

The solid-state studies showed that Risperidone was comparatively stable to the effect of temperature. When the drug powder was exposed to dry heat at 80°C for 24 hr 30.09% degradation was observed with corresponding rise to degradants products at RRT 2.75.

#### 3.3.5. Photolytic Conditions

No major degradation product was observed after exposure of solid drug kept in UV light. Minor degradants products at RRT 2.76 with 26.62% fall in Risperidone after 36 hr.

#### 3.3.6. Assay

**Figure 2:**
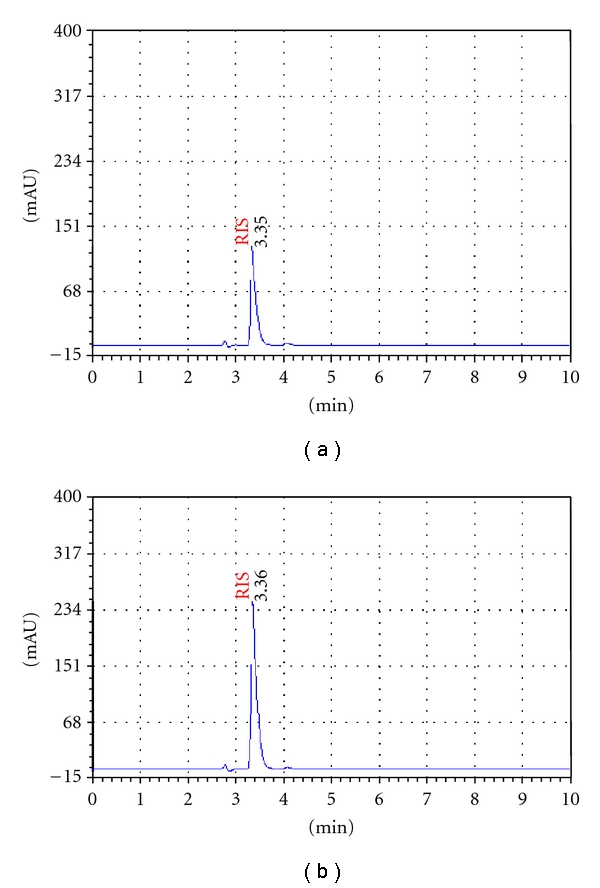
Resultant HPLC chromatograms following the analysis of a standard solution of Risperidone 20 *μ*g/mL (a) and Risperidone tablets (2 mg) (b).

The proposed method was applied to the determination of Risperidone in risperdal. A typical chromatogram obtained following the assay of Risperidone tablets is depicted in Figure 2. The result of these assays yielded 99.88%  (%R.S.D. = 1.31%) of labelled claim for the tablets.

## 4. Conclusions

A validated stability-indicating HPLC analytical method has been developed for the determination of Risperidone in API. The results of stress testing undertaken according to the International Conference on Harmonization (ICH) guidelines reveal that the method is selective and stability indicating. The proposed method is simple, accurate, precise, specific, and has the ability to separate the drug from degradation products and excipients found in the tablet dosage forms. The method is suitable for the routine analysis of Risperidone in either bulk API powder or in pharmaceutical dosage forms. The simplicity of the method allows its application in laboratories that lack sophisticated analytical instruments such as LC–MS or GC–MS. These methods are complicated and costly rather than a simple HPLC-UV method.

## Figures and Tables

**Table 1:** Precision study.

Drug	Concentration (*μ*g/mL)	Intraday precision Calculated concentration ± S.D. (*μ*gmL^−1^), R.S.D.	Interday precision calculated concentration ± S.D. (*μ*gmL^−1^), R.S.D.
Risperidone	20	19.88 ± 0.20, 1.04	20.04 ± 0.24, 1.22
	30	29.85 ± 0.26, 0.89	29.82 ± 0.26, 0.88
	40	39.92 ± 0.21, 0.52	39.87 ± 0.27, 0.68

**Table 2:** Recovery study.

Label claim Mg/tablet	Amount added %	Total amount added (mg)	Amount recovered* (mg) ± SD	% Recovery ± SD (*n* = 3)
Risperidone	50	1	0.99 ± 0.02	99.66 ± 1.79
2	100	2	2.01 ± 0.02	100.21 ± 1.11
	150	3	3.01 ± 0.04	100.31 ± 1.46

*Mean ± %SD for three determinations.

**Table 3:** System suitability parameters.

System suitability parameters	Risperidone
Retention times (RT)	3.34
Theoretical plates (*N*)	3232.65
Tailing factor (AS)	1.83

**Table 4:** Summary of forced degradation results.

Sr. no.	Stress/exposure condition	Drug remained/20 *μ*g/mL ± SD (*n* = 3)	Retention time of degradants	% Recovery	% Degradation
(1)	Acid degradation	14.62 ± 0.16	4.20	73.10	26.89
(2)	Base degradation	16.49 ± 0.16	2.62	82.46	17.53
(3)	Oxidation	6.29 ± 0.08	2.67	31.45	68.54
(4)	Thermal degradation	13.98 ± 0.11	2.75	69.90	30.09
(5)	Photo degradation	14.67 ± 0.12	2.76	73.37	26.62
